# Association of Paternal Age Alone and Combined with Maternal Age with Perinatal Outcomes: A Prospective Multicenter Cohort Study in China

**DOI:** 10.1007/s44197-023-00175-4

**Published:** 2024-01-08

**Authors:** Shaohua Yin, Yubo Zhou, Cheng Zhao, Jing Yang, Pengbo Yuan, Yangyu Zhao, Hongbo Qi, Yuan Wei

**Affiliations:** 1 Department of Obstetrics and Gynecology, National Clinical Research Center for Obstetrical and Gynecology, National Center for Healthcare Quality Management in Obstetrics, Peking University Third Hospital, Haidian District, 49 North Garden Rd., Beijing, 100191 China; 2 National Clinical Research Center for Obstetrical and Gynecology, Peking University Third Hospital, Beijing, 100191 China; 3 Institute of Reproductive and Child Health/National Health Commission Key Laboratory of Reproductive Health, Peking University Health Science Center, Beijing, 100191 China; 4 Department of Epidemiology and Biostatistics, School of Public Health, Peking University Health Science Center, Beijing, 100191 China; 5 Department of Obstetrics, Women and Children’s Hospital of Chongqing Medical University, No. 120 Longshan Road, Yubei District, Chongqing, 400021 China

**Keywords:** Paternal age, Maternal age, Perinatal outcomes, Joint association, Cohort study

## Abstract

Maternal and paternal age at birth is increasing globally. Maternal age may affect perinatal outcomes, but the effect of paternal age and its joint effect with maternal age are not well established. This prospective, multicenter, cohort analysis used data from the University Hospital Advanced Age Pregnant Cohort Study in China from 2016 to 2021, to investigate the separate association of paternal age and joint association of paternal and maternal age with adverse perinatal outcomes. Of 16,114 singleton deliveries, mean paternal and maternal age (± SD) was 38.0 ± 5.3 years and 36.0 ± 4.1 years. In unadjusted analyses, older paternal age was associated with increased risks of gestational diabetes mellitus (GDM), hypertensive disorders of pregnancy, preeclampsia, placenta accreta spectrum disorders, placenta previa, cesarean delivery (CD), and postpartum hemorrhage, preterm birth (PTB), large-for-gestational-age, macrosomia, and congenital anomaly, except for small-for-gestational-age. In multivariable analyses, the associations turned to null for most outcomes, and attenuated but still significant for GDM, CD, PTB, and macrosomia. As compare to paternal age of < 30 years, the risks in older paternal age groups increased by 31–45% for GDM, 17–33% for CD, 32–36% for PTB, and 28–31% for macrosomia. The predicted probabilities of GDM, placenta previa, and CD increased rapidly with paternal age up to thresholds of 36.4–40.3 years, and then plateaued or decelerated. The risks of GDM, CD, and PTB were much greater for pregnancies with younger paternal and older maternal age, despite no statistical interaction between the associations related to paternal and maternal age. Our findings support the advocation that paternal age, besides maternal age, should be considered during preconception counseling.

**Trial Registration** NCT03220750, Registered July 18, 2017—Retrospectively registered, https://classic.clinicaltrials.gov/ct2/show/NCT03220750.

**Supplementary Information:**

The online version contains supplementary material available at 10.1007/s44197-023-00175-4.

## Introduction

The past decades have seen a remarkable increase in proportion of live births to parents of advanced age in many countries, especially among fathers aged 35–39 years [1, 2]. Between 1995 and 2015, the number of live births per 1000 fathers aged 35–39 years raised from 50.4 to 69.1 in the US [2], and from 57.5 to 83.6 in the UK [1]. Although the corresponding figure was unknown in China, the average age of marriages for men increased from 24 years in 1990 to 29 years in 2020 [3], approaching the 30 years for men in the US in 2020 [4].

Most previous studies focused on the effects of paternal age on offspring outcomes [5–7], but data about the effects on maternal outcomes are sparse and equivocal [6, 8, 9]. For example, one study in Israel found that paternal age of 35 years or older was associated with a higher risk of preeclampsia compared with paternal age of 25–34 years [8], whereas a study in the US did not observe the association [6], despite both studies conducted with large sample sizes. Furthermore, there was still no biological plausible threshold to define advanced paternal age. Defining reliable threshold requires understanding of whether relationship between paternal age and risk of outcome is linear or non-linear, but such study is lacking. Although maternal and paternal age have been shown to independently affect perinatal outcomes [6, 8, 10], their joint effects were still unclear. There are two studies only reported the joint effects for individual outcomes and the results were inconsistent [11, 12]. One study in the Texas–Mexico border found that paternal age of 35 years or older combined with advanced maternal age was associated with an increased risk of gestational hypertensive disorders, compared to paternal and maternal age younger than 35 years [12]. While the other study focusing on low birth weight, and preterm birth did not found any significant associations [11].

Using data from the University Hospital Advanced Age Pregnant (UNIHOPE) Cohort in China, we aimed to examine the separate association of paternal age, and joint associations of paternal and maternal age with adverse perinatal outcomes in mothers and offspring, and attempted to identify potential threshold for defining advanced paternal age by examining the linear or non-linear relationships between paternal age and adverse outcomes.

## Methods

### Study design and participants

The multicenter prospective UNIHOPE cohort was conducted in China from July 2016 to June 2021, aiming to explore potential predictors of adverse pregnancy outcomes among women of advanced age (ClinicalTrails.gov: NCT03220750) [13]. The cohort was conducted in eight public referral hospitals located in Beijing, Shanghai, Guangzhou, Shenyang, Wuhan, Chongqing, and Chengdu, scattered through the eastern, central, and western regions of China. Pregnant women were enrolled before 14 weeks of gestation, and followed up at 24–28 weeks, 32–34 weeks of gestation, delivery, and 6–12 weeks postpartum, by obstetrician or nurse in the hospitals. Information on sociodemographic characteristics and lifestyle (maternal and paternal), medical and reproductive history, prenatal care of the current pregnancy, and pregnancy outcomes was collected at enrollment and subsequent follow-ups using a structured questionnaire.

**Fig. 1:**
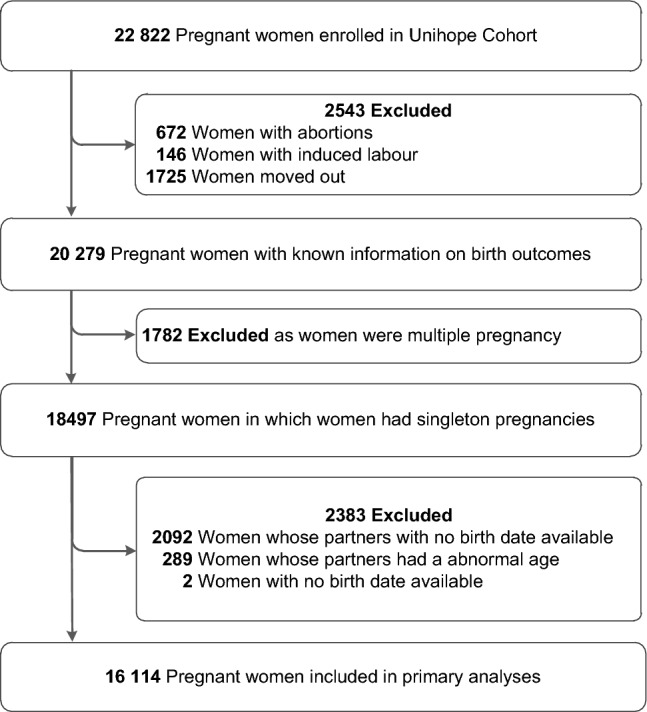
Flow chart of participants selection

Pregnant women were eligible in the UNIHOPE cohort if they attended regular antenatal care and delivered at the included hospitals. Women would be excluded if they had mental disorders or had no ability to provide informed consent. Initially, a total of 22,822 pregnant women were enrolled in the UNIHOPE cohort. For this study, 6708 women who were ended with spontaneous or induced abortions (*n* = 818), moved out (*n* = 1725), had multiple pregnancies (*n* = 1782), had no birthdate (*n* = 2), or whose partners had no birthdate (*n* = 2092) or abnormal age (< 20 or > 70 years, *n* = 289) were excluded. Finally, 16,114 pregnant women remained in the analysis (Fig. 1).

### Exposures and covariates

The main exposure was paternal age, which was defined as the male partner’s age at the time of conception, calculating as subtracting the partner’s birthdate from the last menstrual period date. Paternal age was used continuously or categorically (< 30, 30–34, 35–39, 40–44, and ≥ 45 years) in this analysis, as appropriate. Maternal age was calculated as subtracting the maternal birthdate from the date of delivery, and categorized into four groups: < 35, 35–39, 40–44, and ≥ 45 years.

Other covariates included paternal occupation, alcohol consumption and smoking, as well as maternal ethnicity, education, occupation, alcohol consumption and smoking within 6 months prior to pregnancy, pre-pregnancy body mass index (BMI), gestational age at enrolment, illness before pregnancy, parity, method of conception, annual household income, and delivery year. Gestational age at enrollment was determined by the last menstrual period for women with regular menstrual cycles or by ultrasound for others.

### Outcomes of interest

Interested maternal outcomes included: (1) gestational diabetes mellitus (GDM), defined as a fasting plasma glucose ≥ 5.1 mmol/L or an OGTT 1 h plasma glucose ≥ 10.0 mmol/L, or an OGTT 2 h plasma glucose ≥ 8.5 mmol/L, that occurs or is first diagnosed during pregnancy [14]; (2) hypertensive disorders of pregnancy (HDP), defined as a spectrum of conditions including preeclampsia, preeclampsia superimposed on chronic hypertension, gestational hypertension, and chronic hypertension; (3) preeclampsia, defined as new-onset blood pressures ≥ 140/90 mmHg after 20 weeks of gestation and combined with albuminuria ≥ 0.3 g [15]; (4) placenta accreta spectrum (PAS) disorders, characterized as abnormal trophoblast invasion of part or all of the placenta into the myometrium of the uterine wall [16]; (5) placenta previa (PP), defined as placenta complete or partial covering the internal orifice of cervix; (6) cesarean delivery (CD), identified by inpatient medical records; and (7) postpartum hemorrhage (PPH), defined as a loss of ≥ 500 ml of blood after vaginal delivery or ≥ 1000 ml after CD within 24 h of birth [17].

Interest offspring outcomes included: (1) preterm birth (PTB) defined as a live birth occurred before 37 completed gestational weeks; (2) large-for-gestational-age (LGA) defined as birthweight of > 90th percentile for gestational age of a China reference population [18]; (3) small-for-gestational-age (SGA) defined as birthweight of < 10th percentile for gestational age; (4) macrosomia defined as a newborn with a birthweight ≥ 4000 g; and (5) congenital anomaly defined as congenital structural anomalies for fetus or infants, such as congenital heart defect, Down’s syndrome, polydactylism, and cleft palate.

### Statistical analysis

Differences in paternal and maternal characteristics across paternal age groups were determined using Chi-square tests for categorical variables and one-way analysis of variance with Dunnett corrections for multiple comparisons for continuous variables.

Univariate and multivariable log-binomial regression models were performed to estimate relative risks (RRs) and 95% confidence intervals (95% CIs) of adverse outcomes according to paternal age. Because maternal age was associated with increased risks of maternal and offspring outcomes [10, 19], the joint effects of paternal and maternal age on those adverse outcomes were examined. In analysis of joint effects, pregnant women were categorized into 15 strata according to paternal age (< 30, 30–34, 35–39, 40–44, ≥ 45 years) and maternal age (< 35, 35–39, ≥ 40 years), and women aged < 35 years and paternal age < 30 years were used as the reference category. Whether the associations between paternal age and outcomes differed across maternal age was evaluated by adding a multiplicative interaction term into multivariable models. Additive interaction between paternal age and maternal age was also examined by using Delta method to calculate the relative excess risk due to interaction (RERI) and 95% CI [20].

Multivariable logistic regression with restricted cubic spline were performed to assess the relationships of paternal age with adverse outcomes. The number of knots (between 3 and 7) was determined based on the lowest Akaike information criterion. Adjusted odds of each outcome in 1-year increments of paternal age from 20 to 70 years were calculated, and then transformed to predicted probabilities. The predicted probabilities with 95% confidence intervals (95% CI) were plotted for paternal age to display the relationships visually. If non-linear relationship was observed, segmented model was then used to identify the inflection point [21].

In multivariable analysis, the adjusted covariates included paternal occupation, smoking and alcohol consumption, as well as maternal age, ethnicity, education, occupation, annual household income, smoking, and alcohol consumption within 6 months prior to pregnancy, pre-pregnancy BMI, gestational age at enrolment, illness before pregnancy, parity, method of conception, and delivery year. Missing covariate values were classified into a new category in a dummy variable (0 = not missing; 1 = missing) and included in the models.

To assess the robustness of the results, sensitivity analyses were conducted by using multiply imputed missing values for covariates, or by excluding women who conceived with ART. Additionally, ordinal regression models were used to assess the associations of the paternal and maternal age with perinatal outcomes.

Statistical analyses were performed using SAS software, version 9.4 (SAS Institute, Inc). A 2-sided *P* < 0.05 was deemed statistically significant.

## Results

Among 16,114 deliveries included in the analysis, the average paternal age was 38.0 (SD 5.3) years, and the average maternal age was 36.0 (SD 4.1) years. Maternal age increased as increasing paternal age (Table 1). Both paternal and maternal characteristics differed across paternal age groups (Table 1). As compared with paternal age < 30 years group, pregnant women and their partners in older paternal age groups were more likely to be office workers and drink alcohol, and pregnant women were more likely to be overweight or obesity before pregnancy, have illness before pregnancy, to be multipara, and utilize assisted reproductive technology (ART), have higher education level and annual household income (Table 1).

**Table 1:** Paternal and maternal characteristics, overall and by paternal age group

Characteristics	Total	Paternal age, years					*P* value^a^
		< 30	30–34	35–39	40–44	≥ 45	
Participants	16 114	1160 (7.2)	2756 (17.1)	7083 (44.0)	3691 (22.9)	1424 (8.8)	
Paternal characteristics							
Paternal age, year (mean ± SD)	38.0 ± 5.3	27.7 ± 1.9	32.8 ± 1.4	37.6 ± 1.3	42.1 ± 1.4	48.2 ± 3.3	
Occupation							< 0.001
Office worker	7492 (46.5)	412 (35.5)	1188 (43.1)	3451 (48.7)	1785 (48.4)	656 (46.1)	
Labor worker	2449 (15.2)	226 (19.5)	453 (16.4)	1037 (14.6)	497 (13.5)	236 (16.6)	
Others	6148 (38.2)	518 (44.7)	1114 (40.4)	2586 (36.5)	1400 (37.9)	530 (37.2)	
Unknown	25 (0.2)	4 (0.3)	1 (0)	9 (0.1)	9 (0.2)	2 (0.1)	
Smoking status							< 0.001
Non-smoker	10 819 (67.1)	757 (65.3)	1797 (65.2)	4908 (69.3)	2476 (67.1)	881 (61.9)	
Smoker	4551 (28.2)	359 (30.9)	814 (29.5)	1891 (26.7)	1026 (27.8)	461 (32.4)	
Unknown	744 (4.6)	44 (3.8)	145 (5.3)	284 (4.0)	189 (5.1)	82 (5.8)	
Alcohol consumption							< 0.001
Non-drinker	10 484 (65.1)	796 (68.6)	1828 (66.3)	4648 (65.6)	2353 (63.7)	859 (60.3)	
Drinker	4863 (30.2)	319 (27.5)	780 (28.3)	2136 (30.2)	1145 (31.0)	483 (33.9)	
Unknown	767 (4.8)	45 (3.9)	148 (5.4)	299 (4.2)	193 (5.2)	82 (5.8)	
Maternal characteristics							
Maternal age, year (mean ± SD)	36.0 ± 4.1	28.2 ± 3.8	32.7 ± 3.4	36.4 ± 2.2	38.6 ± 2.4	39.9 ± 3.4	< 0.001
< 35	3555 (22.1)	1039 (89.6)	1729 (62.7)	686 (9.7)	73 (2.0)	28 (2.0)	
35–39	10 016 (62.2)	107 (9.2)	972 (35.3)	6000 (84.7)	2309 (62.6)	628 (44.1)	
≥ 40	2543 (15.8)	14 (1.2)	55 (2.0)	397 (5.6)	1309 (35.5)	768 (54.0)	
Ethnicity							< 0.001
Han	15 361 (95.3)	1096 (94.5)	2573 (93.4)	6771 (95.6)	3546 (96.1)	1375 (96.6)	
Other	737 (4.6)	62 (5.3)	179 (6.5)	307 (4.3)	141 (3.8)	48 (3.4)	
Unknown	16 (0.1)	2 (0.2)	4 (0.1)	5 (0.1)	4 (0.1)	1 (0.1)	
Education							< 0.001
Primary or less	129 (0.8)	4 (0.3)	13 (0.5)	55 (0.8)	44 (1.2)	13 (0.9)	
Secondary	821 (5.1)	48 (4.1)	103 (3.7)	310 (4.4)	253 (6.9)	107 (7.3)	
High school or above	15 111 (93.8)	1104 (95.2)	2633 (95.5)	6695 (94.5)	3381 (91.6)	1298 (88.5)	
Unknown	53 (0.3)	4 (0.3)	7 (0.3)	23 (0.3)	13 (0.4)	48 (3.3)	
Occupation							< 0.001
Office worker	7492 (46.5)	412 (35.5)	1188 (43.1)	3451 (48.7)	1785 (48.4)	656 (46.1)	
Labor worker	2449 (15.2)	226 (19.5)	453 (16.4)	1037 (14.6)	497 (13.5)	236 (16.6)	
Others	6148 (38.2)	518 (44.7)	1114 (40.4)	2586 (36.5)	1400 (37.9)	530 (37.2)	
Unknown	25 (0.2)	4 (0.3)	1 (0)	9 (0.1)	9 (0.2)	2 (0.1)	
Annual household income							< 0.001
Low	6441 (40.0)	269 (23.2)	920 (33.4)	3100 (43.8)	1587 (43.0)	565 (39.7)	
Middle	1670 (10.4)	129 (11.1)	286 (10.4)	700 (9.9)	398 (10.8)	157 (11.0)	
High	7190 (44.6)	705 (60.8)	1386 (50.3)	2984 (42.1)	1502 (40.7)	613 (43.0)	
Unknown	813 (5.0)	57 (4.9)	164 (6.0)	299 (4.2)	204 (5.5)	89 (6.3)	
Pre-pregnancy BMI, kg/m^2^ (mean ± SD)^b^	22.3 ± 3.4	21.2 ± 3.2	22.2 ± 3.7	22.3 ± 3.3	22.6 ± 3.2	22.6 ± 3.3	< 0.001
Underweight	1320 (8.2)	192 (16.6)	285 (10.3)	548 (7.7)	225 (6.1)	70 (4.9)	
Normal weight	11 960 (74.2)	831 (71.6)	1985 (72.0)	5320 (75.1)	2738 (74.2)	1086 (76.3)	
Overweight	2265 (14.1)	99 (8.5)	369 (13.4)	977 (13.8)	596 (16.1)	224 (15.7)	
Obesity	396 (2.5)	20 (1.7)	81 (2.9)	173 (2.4)	89 (2.4)	33 (2.3)	
Unknown	173 (1.1)	18 (1.6)	36 (1.3)	65 (0.9)	43 (1.2)	11 (0.8)	
Gestational age at enrolment, weeks (mean ± SD)	12.6 ± 4.5	12.1 ± 3.6	12.2 ± 4.8	12.6 ± 4.5	12.8 ± 4.6	12.8 ± 4.4	< 0.001
Smoking status within 6 months prior to pregnancy							0.200
Non-smoker	15 639 (97)	1122 (96.7)	2656 (96.4)	6891 (97.3)	3589 (97.2)	1378 (96.8)	
Smoker	253 (1.6)	22 (1.9)	57 (2.1)	105 (1.5)	46 (1.2)	23 (1.6)	
Unknown	225 (1.4)	16 (1.4)	43 (1.6)	87 (1.2)	56 (1.5)	23 (1.6)	
Alcohol consumption within 6 months prior to pregnancy							< 0.001
Non-drinker	14 883 (92.4)	1096 (94.5)	2559 (92.9)	6573 (92.8)	3372 (91.4)	1283 (90.1)	
Drinker	968 (6.0)	43 (3.7)	146 (5.3)	405 (5.7)	255 (6.9)	119 (8.4)	
Unknown	263 (1.6)	21 (1.8)	51 (1.9)	105 (1.5)	64 (1.7)	22 (1.5)	
Diseases before pregnancy^c^	3754 (23.3)	163 (14.1)	596 (21.6)	1709 (24.1)	865 (23.4)	421 (29.6)	< 0.001
Parity							< 0.001
Nullipara	2759 (17.1)	605 (52.2)	867 (31.5)	863 (12.2)	295 (8.0)	129 (9.1)	
Multipara	13 355 (82.9)	555 (47.8)	1889 (68.5)	6220 (87.8)	3396 (92.0)	1295 (90.9)	
Method of conception							< 0.001
Spontaneous	13 576 (84.2)	1095 (94.4)	2387 (86.6)	5914 (83.5)	3070 (83.2)	1110 (77.9)	
Assisted reproductive technology	2292 (14.3)	31 (2.7)	289 (10.5)	1080 (15.3)	589 (15.9)	303 (21.3)	
Unknown	246 (1.5)	34 (2.9)	80 (2.9)	89 (1.3)	32 (0.9)	11 (0.8)	

*SD* standard deviation, *BMI* body mass index

Values are numbers (percentages) unless stated otherwise. Percentages have been rounded and may not total 100

^a^Differences in paternal and maternal characteristics across paternal age groups were determined using Chi-square tests for categorical variables and one-way analysis of variance for continuous variables

^b^Body mass index is weight (kg) divided by height squared (m^2^)

^c^Diseases before pregnancy was defined as the presence of at least one of the 8 diseases recorded before pregnancy, including heart disease, renal disease, hepatic disease, immune system disease, reproductive system disease, thyroid disease, hypertension, and diabetes

In the unadjusted analysis, pregnant women with older partners were associated with higher risks of all adverse maternal outcomes, as compared with those with partners’ age of < 30 years, and the difference was not significant after adjusting for multiple comparisons (Table 2**, **Supplementary Table 1). In the analyses adjusted for maternal age and other confounders, the associations turned to null for most outcomes, and attenuated but still significant for GDM and CD. As compared to pregnant women with partners aged < 30 years, those with partners aged 40–44 years (adjusted RR [aRR] 1.45, 95% CI 1.20–1.76) and ≥ 45 years (aRR 1.44, 95% CI 1.17–1.76) had the highest risk of GDM, followed by age 35–39 years (aRR 1.37, 95% CI 1.14–1.64) and 30–34 years (aRR 1.31, 95% CI 1.10–1.56). The risk of CD was highest in women with partners aged ≥ 45 years (aRR 1.33, 95% CI 1.16–1.52), followed by age 40–44 years (aRR 1.26, 95% CI 1.11–1.43), 35–39 years (aRR 1.27, 95% CI 1.12–1.42), and 30–34 years (aRR 1.17, 95% CI 1.04–1.31). In addition, women with partners aged 40–44 years were also had higher risks of PP (aRR 1.49, 95% CI 1.01–2.18) and PPH (aRR 1.48, 95% CI 1.01–2.17), as compared with those with partners aged < 30 years.

**Table 2:** Associations of paternal age with maternal outcomes

Maternal outcomes	Case (%)	Paternal age (years)				
		< 30	30–34	35–39	40–44	≥ 45
Gestational diabetes mellitus	4373 (27.1)					
Unadjusted		Reference	1.47 (1.24–1.74)	1.80 (1.55–2.11)	2.12 (1.81–2.49)	2.22 (1.86–2.63)
Adjusted		Reference	**1.31 (1.10–1.56)**	**1.37 (1.14–1.64)**	**1.45 (1.20–1.76)**	**1.44 (1.17–1.76)**
Hypertensive disorders of pregnancy	1630 (10.2)					
Unadjusted		Reference	1.57 (1.19–2.06)	1.90 (1.47–2.45)	2.01 (1.54–2.61)	2.24 (1.68–2.98)
Adjusted		Reference	1.06 (0.79–1.41)	0.94 (0.70–1.27)	0.89 (0.65–1.21)	0.94 (0.67–1.32)
Preeclampsia	754 (4.7)					
Unadjusted		Reference	1.95 (1.25–3.03)	2.33 (1.54–3.52)	2.61 (1.71–3.98)	2.68 (1.70–4.23)
Adjusted		Reference	1.33 (0.84–2.10)	1.21 (0.75–1.93)	1.20 (0.73–1.95)	1.18 (0.70–1.99)
Placenta accreta spectrum disorder	1287 (8.0)					
Unadjusted		Reference	1.57 (1.08–2.30)	2.92 (2.07–4.12)	3.48 (2.45–4.95)	3.43 (2.36–4.98)
Adjusted		Reference	1.11 (0.75–1.65)	1.15 (0.77–1.71)	1.27 (0.84–1.91)	1.27 (0.82–1.96)
Placenta previa	1329 (8.3)					
Unadjusted		Reference	1.82 (1.28–2.59)	2.64 (1.90–3.67)	3.12 (2.23–4.35)	2.94 (2.05–4.21)
Adjusted		Reference	1.33 (0.92–1.92)	1.35 (0.93–1.95)	**1.49 (1.01–2.18)**	1.40 (0.92–2.11)
Cesarean delivery	9652 (59.9)					
Unadjusted		Reference	1.28 (1.15–1.43)	1.69 (1.53–1.87)	1.80 (1.62–1.99)	1.92 (1.71–2.14)
Adjusted		Reference	**1.17 (1.04–1.31)**	**1.27 (1.12–1.42)**	**1.26 (1.11–1.43)**	**1.33 (1.16–1.52)**
Postpartum hemorrhage	1278 (7.9)					
Unadjusted		Reference	1.95 (1.38–2.76)	2.42 (1.75–3.35)	2.86 (2.06–3.98)	2.63 (1.84–3.77)
Adjusted		Reference	1.26 (0.88–1.80)	1.36 (0.94–1.95)	**1.48 (1.01–2.17)**	1.36 (0.90–2.04)
Adverse maternal outcome	12,111 (75.2)					
Unadjusted		Reference	1.28 (1.17–1.41)	1.54 (1.42–1.68)	1.63 (1.49–1.78)	1.67 (1.51–1.85)
Adjusted		Reference	**1.17 (1.07–1.29)**	**1.24 (1.12–1.37)**	**1.25 (1.12–1.39)**	**1.27 (1.12–1.42)**

Bold values indicate significant *P*-values

Data are relative risk with 95% confidence intervals. Adjusted for paternal factors including occupation, smoking and alcohol consumption, and maternal factors including delivery year, age, ethnicity, education, occupation, annual household income, gestational age at enrolment, pre-pregnancy BMI, smoking and alcohol consumption within 6 months prior to pregnancy, parity, and method of conception

After accounting for multiple comparisons in unadjusted analyses, older paternal age was associated with increased risks of adverse offspring outcomes except for SGA (Table 3**, **Supplementary Table 1). In analyses adjusted for maternal age and other confounders, the associations turned to null for most outcomes, and attenuated but still significant for PTB and macrosomia (Table 3). As compare to neonates born to fathers aged < 30 years, those born to fathers aged 30–34 years (aRR 1.32, 95% CI 1.00–1.74]) and 40–44 years (aRR 1.36, 95% CI 1.01–1.84) had a greater risk of PTB; and neonates born to fathers aged 35–39 years (aRR 1.31, 95% CI 1.05–1.63), 40–44 years (aRR 1.28, 95% CI 1.02–1.61), ≥ 45 years (aRR 1.31, 95% CI 1.01–1.68) had a greater risk of macrosomia.

**Table 3:** Associations of paternal age with offspring outcomes

Offspring outcomes	Case (%)	Paternal age (years)				
		< 30	30–34	35–39	40–44	≥ 45
Preterm birth	1815 (11.3)					
Unadjusted		Reference	1.57 (1.20–2.05)	2.00 (1.56–2.56)	2.29 (1.78–2.95)	2.00 (1.51–2.65)
Adjusted		Reference	**1.32 (1.00–1.74)**	1.28 (0.96–1.71)	**1.36 (1.01–1.84)**	1.29 (0.92–1.79)
Large for gestational age^a^	2608 (19.0)					
Unadjusted		Reference	1.21 (0.99–1.49)	1.48 (1.23–1.78)	1.49 (1.23–1.81)	1.45 (1.17–1.80)
Adjusted		Reference	1.08 (0.87–1.34)	1.23 (0.99–1.54)	1.22 (0.97–1.55)	1.22 (0.94–1.58)
Small for gestational age^a^	903 (6.6)					
Unadjusted		Reference	0.95 (0.71–1.28)	1.02 (0.78–1.33)	0.92 (0.69–1.22)	0.84 (0.60–1.18)
Adjusted		Reference	0.99 (0.72–1.36)	1.08 (0.77–1.51)	0.99 (0.69–1.41)	0.94 (0.62–1.42)
Macrosomia	2726 (16.9)					
Unadjusted		Reference	1.26 (1.03–1.54)	1.66 (1.39–2.00)	1.70 (1.41–2.06)	1.69 (1.36–2.09)
Adjusted		Reference	1.13 (0.91–1.39)	**1.31 (1.05–1.63)**	**1.28 (1.02–1.61)**	**1.31 (1.01–1.68)**
Congenital anomaly	564 (3.5)					
Unadjusted		Reference	1.75 (1.05–2.93)	2.31 (1.43–3.73)	2.65 (1.63–4.33)	2.94 (1.75–4.96)
Adjusted		Reference	1.16 (0.68–1.98)	1.02 (0.59–1.76)	1.08 (0.61–1.91)	1.10 (0.60–2.01)
Adverse offspring outcome	5138 (31.9)					
Unadjusted		Reference	1.22 (1.06–1.41)	1.55 (1.36–1.76)	1.59 (1.39–1.82)	1.55 (1.33–1.81)
Adjusted		Reference	1.09 (0.94–1.27)	**1.19 (1.02**–**1.39)**	1.17 (0.99–1.38)	1.16 (0.96–1.39)

Bold values indicate significant *P*-values

Data are relative risk with 95% confidence intervals. Adjusted for paternal factors including occupation, smoking and alcohol consumption, and maternal factors including delivery year, age, ethnicity, education, occupation, annual household income, gestational age at enrolment, pre-pregnancy BMI, smoking and alcohol consumption within 6 months prior to pregnancy, parity, and method of conception

^a^Among a total of 16,114 singleton women analyzed in this study, there were 2416 women (15.0%) with missing information on birth weight, neonatal sex, or gestational age at delivery who were not included when performing the association analyses

When joint effect of paternal and maternal age was assessed, the highest risks of GDM, CD, PTB, and macrosomia were observed in younger paternal age combined with older maternal age (Table 4), despite no statistical interaction between the associations related to paternal and maternal age (*P*_interaction_ > 0.05) (Supplementary Table 2). For example, the highest risks of GDM (aRR 2.89, 95% CI 1.18–7.08) and CD (aRR 2.53, 95% CI 1.45–4.42) were observed in pregnant women aged ≥ 40 years and with partners aged < 30 years, the highest risk of PTB (aRR 2.28, 95% CI 1.15–4.52) in pregnant women aged ≥ 40 years and with partners aged 30–34 years, and the highest risk of macrosomia (aRR 1.51, 95% CI 1.12–2.04) in women aged ≥ 40 years and with partners aged 35–39 years, as compared with women aged < 35 years and with partners aged < 30 years.

**Table 4:** Joint associations of paternal age and maternal age with the adverse perinatal outcome

Maternal age (years)	Paternal age (years)				
	< 30	30–34	35–39	40–44	≥ 45
Gestational diabetes mellitus					
<35	Reference	**1.32 (1.08–1.61)**	**1.46 (1.15–1.85)**	**2.21 (1.43–3.41)**	1.83 (0.90–3.74)
35–39	**1.63 (1.05–2.55)**	**1.91 (1.54–2.36)**	**1.95 (1.62–2.34)**	**2.12 (1.74–2.57)**	**1.95 (1.54–2.46)**
≥40	**2.89 (1.18–7.08)**	**2.85 (1.80–4.50)**	**2.40 (1.86–3.08)**	**2.39 (1.95–2.94)**	**2.53 (2.04–3.14)**
Cesarean delivery					
<35	Reference	**1.18 (1.04–1.35)**	**1.41 (1.22–1.65)**	**1.50 (1.08–2.08)**	1.51 (0.91–2.50)
35–39	**1.35 (1.01–1.79)**	**1.41 (1.22–1.63)**	**1.49 (1.32–1.68)**	**1.49 (1.31–1.69)**	**1.52 (1.30–1.77)**
≥40	**2.53 (1.45–4.42)**	**1.60 (1.14–2.24)**	**1.61 (1.36–1.90)**	**1.67 (1.46–1.92)**	**1.80 (1.56–2.08)**
Preterm birth					
<35	Reference	1.27 (0.93–1.73)	1.24 (0.85–1.79)	0.87 (0.32–2.40)	2.32 (0.84–6.41)
35–39	1.05 (0.47–2.31)	**1.62 (1.15–2.27)**	**1.56 (1.16–2.09)**	**1.76 (1.29–2.39)**	**1.61 (1.10–2.34)**
≥40	1.05 (0.14–7.61)	**2.28 (1.15–4.52)**	**2.11 (1.47–3.04)**	**1.85 (1.34–2.54)**	**1.78 (1.26–2.52)**
Macrosomia					
<35	Reference	1.07 (0.85–1.35)	**1.51 (1.16–1.97)**	1.12 (0.58–2.15)	0.86 (0.27–2.71)
35–39	1.04 (0.58–1.86)	1.27 (0.98–1.65)	**1.33 (1.06–1.65)**	**1.32 (1.05–1.67)**	**1.37 (1.03–1.82)**
≥40	1.23 (0.30–5.00)	1.11 (0.56–2.22)	**1.51 (1.12–2.04)**	**1.46 (1.14–1.87)**	**1.49 (1.14–1.94)**

Bold values indicate significant *P*-values

Data are relative risk with 95% confidence intervals. Adjusted for paternal factors including occupation, smoking and alcohol consumption, and maternal factors including delivery year, ethnicity, education, occupation, annual household income, gestational age at enrolment, pre-pregnancy BMI, smoking and alcohol consumption within 6 months prior to pregnancy, parity, and method of conception

Sensitivity analyses by using multiply imputed missing values for covariates, by excluding women who conceived with ART, or by using ordinal regression models showed similar results (Supplementary Tables 3–11).

**Fig. 2:**
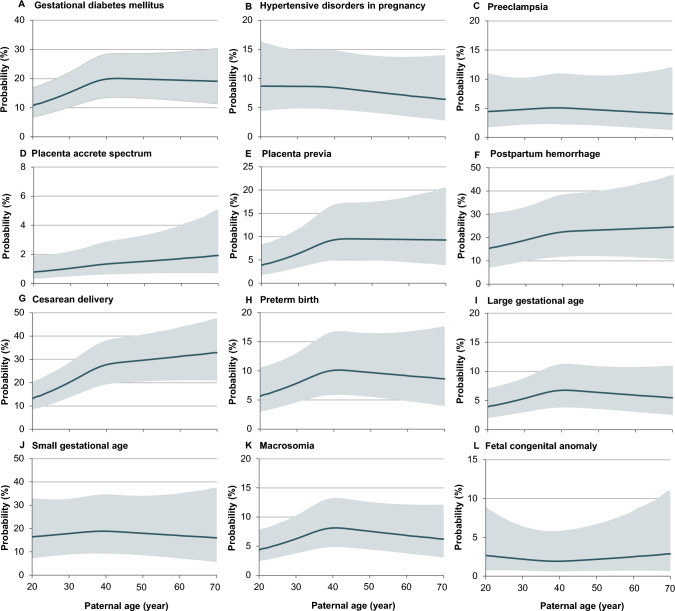
Relationships between paternal age and predicted probabilities of adverse outcomes. Predicted probabilities were transformed from the adjusted odds, which were calculated using multivariable logistic regression models with restricted cubic spline, adjustment for paternal factors including occupation, smoking and alcohol consumption, and maternal factors including delivery year, age, ethnicity, education, occupation, annual household income, gestational age at enrolment, pre-pregnancy BMI, smoking and alcohol consumption within 6 months before pregnancy, parity, and method of conception. Dark gray lines indicate predicted probabilities, and the gray bands represent 95% CIs

Analysis of paternal age as a continuous variable revealed inverted J-shaped relationships with GDM, PP, and CD, linear relationship with PAS, and no relationship with other outcomes (Fig. 2). The predicted probabilities of GDM and PP increased rapidly with paternal age up to thresholds of 38.3 years and 36.4 years then plateaued, and the probabilities of CD increased rapidly until 40.3 years, then decelerated.

## Discussion

This multicenter prospective cohort analysis with 16,114 deliveries showed that older paternal age is associated with increased risks of both maternal and offspring outcomes including GDM, CD, PTB and macrosomia, independently from maternal age and other confounders. However, when taking maternal age into consideration, these risks seem particular high at younger paternal and older maternal age, despite no statistical interaction effect between paternal and maternal age. A critical finding was that there is an inverted J-shaped association of paternal age with risks of GDM, PP and CD, with inflection points around 36–40 years.

Our findings support a large body of literature examining increased risks of some maternal and offspring outcomes following older paternal age, with the highest risks in the oldest ages [6, 9, 22, 23]. A large cohort study using data from 40,529,905 births in the US showed that pregnant women had an increased risk of 16–34% for GDM when their partners aged ≥ 35 years as compared with those partners aged 25–34 years [6]. In our study, the risks of GDM increased by 31–45% in older partner groups as compared with partners aged < 30 years.

Our findings are consistent with previous studies based on a registry of birth data which reported that advancing paternal age was associated with higher risk of CD. A study of 12,589,415 births in the US found that the risks of CD were 24–49% higher in pregnant women with partners aged ≥ 30 years compared with those partners aged 20–29 years [23]. Another study including 15,218 births of nulliparous singleton pregnancies in Lebanon showed that the risk increased by 10–40% in pregnant women with partners aged ≥ 30 years compared with those partners aged < 30 years [22]. In our study, the risks of CD increased by 17–33% in pregnant women with partners aged ≥ 30 years compared with partners aged < 30 years.

Our findings on PTB are in line with several large cohort studies from different nations [6, 24, 25]. For example, a retrospective cohort study in the US showed infants born to fathers aged ≥ 35 years had 6–25% higher risks of preterm as compared with those born to fathers aged 25–34 years [6]. In our study, the risks of PTB increased by 28–36% in infants born to fathers aged ≥ 30 years compared with those born to fathers aged < 30 years.

Studies of paternal age and birth weight showed diverse results [6, 26, 27]. In our study, the risks of macrosomia increased by 28–31% in infants born to fathers aged ≥ 35 years compared with those born to fathers aged < 30 years, similar to the findings from a Korean study [26]. However, a negative relationship between paternal age and birth weight was reported in a US study [26], and no significant association between paternal age and macrosomia was observed in a Turkish study [27].

Studies regarding other maternal complications such as PP and PPH were limited. A retrospective cohort study in the US showed that the incidence of PP increased with increasing partners’ age [9]. In our study, the risks of PP and PPH increased by 48% − 49% in pregnant women with partners aged 40–44 years as compared with partners aged < 30 years. Some studies reported that older paternal age was associated with increased risk of congenital defects [5, 28], which was not observed in our study.

Of note, our study revealed inverted J-shaped associations between paternal age and risks of GDM, PP and CD, with apexes at 36–40 years, thereafter the risks started to plateau or decelerate. These findings indicated that 36–40 years might be the thresholds to define advanced paternal age.

A potential explanation for our findings is the epigenetic link between the aging paternal genome and placental development [29]. RNA sequencing of trophoblast tissues demonstrated that paternal gene expression has a predominant influence on the process of placentation [30]. Male aging-related epigenetic changes (e.g., DNA methylation, chromosomal abnormalities, increased oxidative stress) that occur within spermatocytes lead to enlargement of placenta [31, 32], which was associated with increased risks for adverse maternal outcomes, such as GDM and CD[33, 34]. However, on the other hand, a larger placenta was associated with a greater birthweight and thus a higher risk of macrosomia[35]. Male aging-related epigenetic changes could also result in cell senescence, affect decidua attachment to the placenta, and then increase preterm risk [36, 37]. Additionally, the number of mutations increased with paternal age, partially contributing to the increased preterm risk [38].

Interesting findings were observed in the joint association of paternal and maternal age with adverse perinatal outcomes. We found that pregnancies with younger paternal age combining with older maternal age had greater risks of adverse outcomes including GDM, CD, and PTB. The joint effects of younger paternal and older maternal age could be due to economic and psychological disadvantages that pregnant women suffer from families with younger male partners [39]. However, we have to admit that we did not observe the statistical interaction effect between paternal and maternal age. Further research is needed to determine their joint effects on perinatal outcomes.

Our study has strengths. First, this is the largest multicenter prospective cohort study to date examining adverse outcomes in mothers and offspring in the context of increasing paternal and maternal age in China. Second, this is the first study assessing whether clinical threshold identifying advanced paternal age exists. Third, this study prospectively collected data including parental baseline characteristics, maternal sociodemographic characteristics, medical conditions of prior or current pregnancy, which increased the internal validity of our estimates.

Our study also has several limitations. First, our study population was overwhelmingly recruited from public referral hospitals where women are more likely to be complicated pregnancy, possibly leading to overestimated effects of older paternal age on adverse outcomes. Second, due to insufficient sample size for < 25 and > 50 years paternal age groups, their associations with perinatal outcomes could not be reliably estimated. Third, this study had limited statistical power to detect the differences in risks of some outcomes across paternal age groups, especially for the 30–34 group. Fourth, the unmeasured paternal disease history might introduce potential bias into the associations, and confounding effect of maternal age might not fully adjust. Finally, caution is needed when generalizing the threshold of paternal age derived from this study which warrant to be verified using data from different countries.

## Conclusions

Our findings indicated that older paternal age is independently associated with greater risks of GDM, CD, PTB, and macrosomia, and the risks seem particularly pronounced at pregnancies with younger paternal but older maternal age. An inverted J-shaped association of paternal age with risks of GDM, PP and CD was observed, with the inflection points around 36–40 years. Preconception counseling guidelines might need to be updated to warn potential risks associated with delaying fatherhood, particularly given the still increasing paternal age. Future studies focusing on the health effects of extremely younger or extremely older paternal age are warranted.

## Supplementary Information

Below is the link to the electronic supplementary material.

Supplementary file1 (DOCX 75 KB)

## Data Availability

Data in this study are not publicly available for ethical and legal reasons. Requests for data should be directed to the corresponding author.

## Abbreviations

aRR: Adjusted relative risk
95% CI: 95% Confidence interval
UNIHOPE: University Hospital Advanced Age Pregnant
BMI: Body mass index
GDM: Gestational diabetes mellitus
HDP: Hypertensive disorders of pregnancy
PAS: Placenta accreta spectrum disorders
CD: Cesarean delivery
PPH: Postpartum hemorrhage
LGA: Large-for-gestational-age
SGA: Small-for-gestational-age
RERI: Relative excess risk due to interaction
