# Correction: Association of Paternal Age Alone and Combined with Maternal Age with Perinatal Outcomes: A Prospective Multicenter Cohort Study in China

**DOI:** 10.1007/s44197-025-00367-0

**Published:** 2025-02-06

**Authors:** Shaohua Yin, Yubo Zhou, Cheng Zhao, Jing Yang, Pengbo Yuan, Yangyu Zhao, Hongbo Qi, Yuan Wei

**Affiliations:** 1 Department of Obstetrics and Gynecology, National Clinical Research Center for Obstetrical and Gynecology, National Center for Healthcare Quality Management in Obstetrics, Peking University Third Hospital, Haidian District, 49 North Garden Rd., Beijing, 100191 China; 2 National Clinical Research Center for Obstetrical and Gynecology, Peking University Third Hospital, Beijing, 100191 China; 3 Institute of Reproductive and Child Health/National Health Commission Key Laboratory of Reproductive Health, Peking University Health Science Center, Beijing, 100191 China; 4 Department of Epidemiology and Biostatistics, School of Public Health, Peking University Health Science Center, Beijing, 100191 China; 5 Department of Obstetrics, Women and Children’s Hospital of Chongqing Medical University, No. 120 Longshan Road, Yubei District, Chongqing, 400021 China

**Journal of Epidemiology and Global Health (2024) 14:120-130**

10.1007/s44197-023-00175-4

This article was published with an incorrect grant number in the funding section. The funding number is currently listed as “[2021YFC270150001],” but the correct number should be “[2021YFC27015001].”

The Original Article has been corrected.

