# Supplementary material for: Association of Paternal Age Alone and Combined with Maternal Age with Perinatal Outcomes: A Prospective Multicenter Cohort Study in China
Source: J Epidemiol Glob Health. 2024 Jan 8;14(1):120–30. doi: 10.1007/s44197-023-00175-4 (PMC11043302; doi:10.1007/s44197-023-00175-4)
Supplement: Supplementary file 1 — Supplementary file1 (DOCX 75 KB) [file 44197_2023_175_MOESM1_ESM.docx]

**Association of paternal age alone and combined with maternal age with perinatal outcomes: a prospective multicenter cohort study in China**

Shaohua Yin, PhD, Yubo Zhou, PhD, Cheng Zhao, MD, Jing Yang, MD, Pengbo Yuan, MD, Yangyu Zhao, MD, Hongbo Qi, MD, Yuan Wei, MD

**Appendix**

**Contents**

[Supplementary Table 1. Perinatal outcomes by paternal age group 2](#_Toc138163749)

[Supplementary Table 2. Interactive effects of paternal age and maternal age on perinatal outcomes 3](#_Toc138163750)

[Supplementary Table 3–5. Sensitivity analyses of associations of paternal age with perinatal outcomes using multiple imputation data 5](#_Toc138163751)

[Supplementary Table 6–8. Sensitivity analyses of associations of paternal age with perinatal outcomes among women with spontaneous conception 8](#_Toc138163752)

[Supplementary Table 9–11. Sensitivity analyses of associations of paternal age with perinatal outcomes using ordinal regression 11](#_Toc138163753)

# Supplementary Table 1. Perinatal outcomes by paternal age group ^a^

| **Perinatal outcomes** | **Total (N=16 114)** | **Paternal age, No. (%)** | | | | | ***P*^b^** | ***P*^c^** | ***P^d^*** |
| --- | --- | --- | --- | --- | --- | --- | --- | --- | --- |
|  |  | **<30 (n=1160)** | **30–34 (n=2756)** | **35–39 (n=7083)** | **40–44 (n=3691)** | **≥45 (n=1424)** |  |  |  |
| Gestational diabetes mellitus | 4373 (27.1) | 175 (15.1) | 612 (22.2) | 1928 (27.2) | 1182 (32.0) | 476 (33.4) | <0.001 | <0.001 | <0.001 |
| Hypertensive disorders of pregnancy | 1630 (10.2) | 64 (5.5) | 238 (8.6) | 743 (10.5) | 409 (11.1) | 176 (12.4) | <0.001 | <0.001 | <0.001 |
| Preeclampsia | 754 (4.7) | 24 (2.1) | 111 (4.0) | 341 (4.8) | 199 (5.4) | 79 (5.6) | <0.001 | <0.001 | <0.001 |
| Placenta accreta spectrum disorder | 1287 (8.0) | 34 (2.9) | 127 (4.6) | 606 (8.6) | 377 (10.2) | 143 (10.0) | <0.001 | <0.001 | <0.001 |
| Placenta previa | 1329 (8.3) | 38 (3.3) | 164 (6.0) | 613 (8.7) | 377 (10.2) | 137 (9.6) | <0.001 | <0.001 | <0.001 |
| Cesarean delivery | 9652 (59.9) | 430 (37.1) | 1312 (47.6) | 4437 (62.6) | 2462 (66.7) | 1011 (71.0) | <0.001 | <0.001 | <0.001 |
| Postpartum hemorrhage | 1278 (7.9) | 39 (3.4) | 181 (6.6) | 577 (8.1) | 355 (9.6) | 126 (8.9) | <0.001 | <0.001 | <0.001 |
| Preterm birth | 1815 (11.3) | 68 (5.9) | 254 (9.2) | 831 (11.7) | 495 (13.4) | 167 (11.7) | <0.001 | <0.001 | <0.001 |
| Large for gestational age ^e^ | 2608 (19.0) | 123 (13.6) | 353 (16.4) | 1226 (20.0) | 656 (20.2) | 250 (19.7) | <0.001 | <0.001 | <0.001 |
| Small for gestational age ^e^ | 903 (6.6) | 62 (6.8) | 140 (6.5) | 425 (6.9) | 203 (6.3) | 73 (5.7) | 0.244 | 0.483 | 0.495 |
| Macrosomia | 2726 (16.9) | 126 (10.9) | 376 (13.6) | 1280 (18.1) | 683 (18.5) | 261 (18.3) | <0.001 | <0.001 | <0.001 |
| Congenital anomaly | 564 (3.5) | 18 (1.6) | 75 (2.7) | 254 (3.6) | 152 (4.1) | 65 (4.6) | <0.001 | <0.001 | <0.001 |

^a^ Data are numbers (percentages) unless stated otherwise.

P^b^ and P^c^=P value for linear association. P^d^=P value for quadratic association. P values derived with log-binomial regression models, with maternal age group, maternal age and maternal age² as continuous variables and outcomes as binary.

^e^ Among a total of 16114 singleton women analyzed in this study, there were 2416 women (15.0%) with missing information on birth weight, neonatal sex, or gestational age at delivery who were not included when performing the description and comparison of small for gestational age and large for gestational age.

# Supplementary Table 2. Interactive effects of paternal age and maternal age on perinatal outcomes ^a^

|  | **Maternal age (years)** | | | **RERI (95% CI)** | **P_multiplicative_**  **_interaction_** |
| --- | --- | --- | --- | --- | --- |
| **Paternal age (years)** | **<35** | **35–39** | **≥40** |  |  |
| **Gestational diabetes mellitus** |  |  |  | 0.10 (-0.11–0.31) | 0.388 |
| <30 | Reference | Reference | Reference |  |  |
| 30–34 | 1.21 (0.99–1.48) | 1.18 (0.77–1.81) | 1.02 (0.38–2.74) |  |  |
| 35–39 | **1.31 (1.03–1.67)** | 1.22 (0.81–1.84) | 0.85 (0.34–2.12) |  |  |
| 40–44 | **2.06 (1.32–3.21)** | 1.32 (0.87–2.01) | 0.86 (0.35–2.12) |  |  |
| ≥45 | 1.75 (0.84–3.64) | 1.22 (0.79–1.89) | 0.91 (0.37–2.25) |  |  |
| **Placenta previa** |  |  |  | 0.14 (-22.31–22.58) | 0.167 |
| <30 | Reference | Reference | Reference |  |  |
| 30–34 | 1.26 (0.81–1.95) | 1.18 (0.54–2.56) | 0.93 (0.11–7.88) |  |  |
| 35–39 | 1.18 (0.70–1.97) | 1.16 (0.55–2.46) | 1.14 (0.16–8.40) |  |  |
| 40–44 | **2.80 (1.22–6.41)** | 1.35 (0.63–2.86) | 0.91 (0.13–6.66) |  |  |
| ≥45 | –^b^ | 1.28 (0.58–2.81) | 0.95 (0.13–6.92) |  |  |
| **Cesarean delivery** |  |  |  | 0.11 (-0.12–0.35) | 0.230 |
| <30 | Reference | Reference | Reference |  |  |
| 30–34 | **1.23 (1.07–1.40)** | 1.05 (0.80–1.38) | 0.64 (0.34–1.22) |  |  |
| 35–39 | **1.44 (1.23–1.68)** | 1.10 (0.84–1.42) | 0.66 (0.37–1.17) |  |  |
| 40–44 | **1.41 (1.01–1.98)** | 1.10 (0.85–1.43) | 0.69 (0.39–1.21) |  |  |
| ≥45 | 1.52 (0.92–2.53) | 1.13 (0.86–1.49) | 0.73 (0.42–1.29) |  |  |
| **Postpartum hemorrhage** |  |  |  | -0.02 (-0.23–0.18) | 0.859 |
| <30 | Reference | Reference | Reference |  |  |
| 30–34 | 1.26 (0.83–1.92) | 1.16 (0.50–2.67) | 0.39 (0.07–2.18) |  |  |
| 35–39 | 1.48 (0.92–2.39) | 1.26 (0.56–2.84) | 0.40 (0.09–1.69) |  |  |
| 40–44 | 0.86 (0.26–2.88) | 1.40 (0.62–3.17) | 0.42 (0.10–1.72) |  |  |
| ≥45 | 1.04 (0.14–7.77) | 1.32 (0.57–3.08) | 0.39 (0.09–1.65) |  |  |
| **Preterm birth** |  |  |  | -0.05 (-0.34–0.24) | 0.689 |
| <30 | Reference | Reference | Reference |  |  |
| 30–34 | 1.14 (0.83–1.56) | 1.56 (0.72–3.36) | 2.36 (0.29–18.95) |  |  |
| 35–39 | 1.07 (0.73–1.57) | 1.52 (0.72–3.21) | 2.10 (0.28–15.46) |  |  |
| 40–44 | 0.71 (0.25–2.00) | 1.71 (0.81–3.63) | 1.86 (0.25–13.62) |  |  |
| ≥45 | 2.15 (0.75–6.15) | 1.55 (0.71–3.40) | 1.85 (0.25–13.59) |  |  |
| **Macrosomia** |  |  |  | -0.13 (-0.29–0.02) | 0.704 |
| <30 | Reference | Reference | Reference |  |  |
| 30–34 | 1.13 (0.89–1.44) | 1.20 (0.68–2.12) | 1.01 (0.21–4.75) |  |  |
| 35–39 | **1.62 (1.23–2.13)** | 1.24 (0.72–2.15) | 1.34 (0.32–5.56) |  |  |
| 40–44 | 1.21 (0.62–2.34) | 1.24 (0.71–2.16) | 1.31 (0.32–5.36) |  |  |
| ≥45 | 0.91 (0.29–2.91) | 1.29 (0.72–2.30) | 1.37 (0.33–5.65) |  |  |

^a^ Relative risks were estimated by using multivariable regression, with adjustment for paternal factors including occupation, smoking and alcohol consumption, and maternal factors including delivery year, age, ethnicity, education, occupation, annual household income, gestational age at enrolment, pre-pregnancy BMI, smoking and alcohol consumption within 6 months prior to pregnancy, parity, and method of conception.

^b^ Relative risk with 95% confidence intervals cannot be estimated due to limited available data.

# Supplementary Table 3–5. Sensitivity analyses of associations of paternal age with perinatal outcomes using multiple imputation data

**Supplementary Table 3. Associations of paternal age with maternal outcomes** ^a^

|  | **Paternal age (years)** | | | | |
| --- | --- | --- | --- | --- | --- |
| **Maternal outcomes** | **<30** | **30–34** | **35–39** | **40–44** | **≥45** |
| **Gestational diabetes mellitus** |  |  |  |  |  |
| Unadjusted | Reference | 1.47 (1.24–1.74) | 1.80 (1.55–2.11) | 2.12 (1.81–2.49) | 2.22 (1.86–2.63) |
| Adjusted | Reference | **1.3 (1.09–1.55)** | **1.35 (1.13–1.63)** | **1.44 (1.19–1.75)** | **1.41 (1.15–1.74)** |
| **Hypertensive disorders of pregnancy** |  |  |  |  |  |
| Unadjusted | Reference | 1.57 (1.19–2.06) | 1.90 (1.47–2.45) | 2.01 (1.54–2.61) | 2.24 (1.68–2.98) |
| Adjusted | Reference | 1.04 (0.77–1.39) | 0.92 (0.68–1.24) | 0.87 (0.64–1.19) | 0.90 (0.64–1.26) |
| **Preeclampsia** |  |  |  |  |  |
| Unadjusted | Reference | 1.95 (1.25–3.03) | 2.33 (1.54–3.52) | 2.61 (1.71–3.98) | 2.68 (1.70–4.23) |
| Adjusted | Reference | 1.36 (0.85–2.16) | 1.24 (0.77–2.00) | 1.24 (0.75–2.04) | 1.24 (0.73–2.12) |
| **Placenta accreta** **spectrum disorder** |  |  |  |  |  |
| Unadjusted | Reference | 1.57 (1.08–2.30) | 2.92 (2.07–4.12) | 3.48 (2.45–4.95) | 3.43 (2.36–4.98) |
| Adjusted | Reference | 1.17 (0.78–1.76) | 1.19 (0.79–1.79) | 1.29 (0.85–1.97) | 1.28 (0.82–1.99) |
| **Placenta previa** |  |  |  |  |  |
| Unadjusted | Reference | 1.82 (1.28–2.59) | 2.64 (1.90–3.67) | 3.12 (2.23–4.35) | 2.94 (2.05–4.21) |
| Adjusted | Reference | 1.38 (0.94–2.02) | 1.36 (0.92–2.01) | **1.50 (1.00–2.25)** | 1.41 (0.92–2.17) |
| **Cesarean delivery** |  |  |  |  |  |
| Unadjusted | Reference | 1.28 (1.15–1.43) | 1.69 (1.53–1.87) | 1.80 (1.62–1.99) | 1.92 (1.71–2.14) |
| Adjusted | Reference | **1.16 (1.03–1.30)** | **1.25 (1.11–1.41)** | **1.24 (1.09–1.41)** | **1.31 (1.14–1.50)** |
| **Postpartum hemorrhage** |  |  |  |  |  |
| Unadjusted | Reference | 1.95 (1.38–2.76) | 2.42 (1.75–3.35) | 2.86 (2.06–3.98) | 2.63 (1.84–3.77) |
| Adjusted | Reference | 1.33 (0.92–1.92) | 1.40 (0.96–2.04) | **1.55 (1.05–2.30)** | 1.38 (0.90–2.11) |
| **Adverse maternal outcome** |  |  |  |  |  |
| Unadjusted | Reference | 1.28 (1.17**–**1.41) | 1.54 (1.42**–**1.68) | 1.63 (1.49**–**1.78) | 1.67 (1.51**–**1.85) |
| Adjusted | Reference | **1.18 (1.07–1.30)** | **1.24 (1.11–1.37)** | **1.24 (1.11–1.39)** | **1.26 (1.12–1.43)** |

^a^ Data are relative risk with 95% confidence intervals, estimated by using multiple imputation data. Adjusted for paternal factors including occupation, smoking and alcohol consumption, and maternal factors including delivery year, age, ethnicity, education, occupation, annual household income, gestational age at enrolment, pre-pregnancy BMI, smoking and alcohol consumption within 6 months prior to pregnancy, parity, and method of conception.

**Supplementary Table 4. Associations of paternal age with offspring outcomes** ^a^

|  | **Paternal age (years)** | |  |  |  |
| --- | --- | --- | --- | --- | --- |
| **Offspring outcomes** | **<30** | **30–34** | **35–39** | **40–44** | **≥45** |
| **Preterm birth** |  |  |  |  |  |
| Unadjusted | Reference | 1.57 (1.20–2.05) | 2.00 (1.56–2.56) | 2.29 (1.78–2.95) | 2.00 (1.51–2.65) |
| Adjusted | Reference | 1.29 (0.98–1.72) | 1.27 (0.95–1.71) | **1.33 (1.00–1.81)** | 1.26 (0.90–1.76) |
| **Large for gestational age** ^b^ |  |  |  |  |  |
| Unadjusted | Reference | 1.21 (0.99–1.49) | 1.48 (1.23–1.78) | 1.49 (1.23–1.81) | 1.45 (1.17–1.80) |
| Adjusted | Reference | 1.04 (0.84–1.30) | 1.17 (0.93–1.46) | 1.15 (0.91–1.47) | 1.15 (0.89–1.50) |
| **Small for gestational age** ^b^ |  |  |  |  |  |
| Unadjusted | Reference | 0.95 (0.71–1.28) | 1.02 (0.78–1.33) | 0.92 (0.69–1.22) | 0.84 (0.60–1.18) |
| Adjusted | Reference | 1.01 (0.73–1.39) | 1.09 (0.77–1.52) | 0.99 (0.68–1.42) | 0.99 (0.65–1.51) |
| **Macrosomia** |  |  |  |  |  |
| Unadjusted | Reference | 1.26 (1.03–1.54) | 1.66 (1.39–2.00) | 1.70 (1.41–2.06) | 1.69 (1.36–2.09) |
| Adjusted | Reference | 1.08 (0.87–1.34) | **1.22 (1.03–1.52)** | **1.20 (1.00–1.51)** | **1.22 (1.00–1.57)** |
| **Congenital anomaly** |  |  |  |  |  |
| Unadjusted | Reference | 1.75 (1.05–2.93) | 2.31 (1.43–3.73) | 2.65 (1.63–4.33) | 2.94 (1.75–4.96) |
| Adjusted | Reference | 1.07 (0.62–1.83) | 1.02 (0.58–1.76) | 1.09 (0.61–1.92) | 1.12 (0.61–2.05) |
| **Adverse offspring outcome** |  |  |  |  |  |
| Unadjusted | Reference | 1.22 (1.06–1.41) | 1.55 (1.36–1.76) | 1.59 (1.39–1.82) | 1.55 (1.33–1.81) |
| Adjusted | Reference | 1.06 (0.91–1.24) | **1.15 (1.03–1.35)** | **1.14 (1.00–1.34)** | 1.13 (0.94–1.36) |

^a^ Data are relative risk with 95% confidence intervals, estimated by using multiple imputation data. Adjusted for paternal factors including occupation, smoking and alcohol consumption, and maternal factors including delivery year, age, ethnicity, education, occupation, annual household income, gestational age at enrolment, pre-pregnancy BMI, smoking and alcohol consumption within 6 months prior to pregnancy, parity, and method of conception

^b^ Among a total of 16 114 singleton women analyzed in this study, there were 2416 women (15.0%) with missing information on birth weight, neonatal sex, or gestational age at delivery who were not included when performing the association analyses.

**Supplementary Table 5. Joint associations of paternal age and maternal age with the adverse perinatal outcome** ^a^

|  | **Paternal age (years)** | | | | |
| --- | --- | --- | --- | --- | --- |
| **Maternal age (years)** | **<30** | **30–34** | **35–39** | **40–44** | **≥45** |
| **Gestational diabetes mellitus** |  |  |  |  |  |
| <35 | Reference | **1.31 (1.07–1.60)** | **1.44 (1.14–1.83)** | **2.18 (1.41–3.37)** | 1.96 (0.96–4.00) |
| 35–39 | **1.59 (1.01–2.50)** | **1.86 (1.49–2.32)** | **1.89 (1.56–2.28)** | **2.05 (1.68–2.50)** | **1.90 (1.50–2.41)** |
| ≥40 | **2.88 (1.18–7.06)** | **2.83 (1.78–4.52)** | **2.35 (1.82–3.04)** | **2.34 (1.90–2.88)** | **2.42 (1.94–3.01)** |
| **Cesarean delivery** |  |  |  |  |  |
| <35 | Reference | **1.18 (1.03–1.34)** | **1.37 (1.17–1.60)** | 1.39 (0.99–1.96) | 1.62 (0.98–2.69) |
| 35–39 | 1.27 (0.95–1.70) | **1.37 (1.19–1.59)** | **1.44 (1.27–1.63)** | **1.43 (1.25–1.64)** | **1.47 (1.25–1.72)** |
| ≥40 | **2.43 (1.39–4.24)** | **1.52 (1.07–2.16)** | **1.57 (1.32–1.86)** | **1.61 (1.40–1.85)** | **1.73 (1.50–2.01)** |
| **Preterm birth** |  |  |  |  |  |
| <35 | Reference | 1.26 (0.92–1.73) | 1.20 (0.82–1.76) | 0.87 (0.32–2.42) | 2.58 (0.93–7.13) |
| 35–39 | 1.10 (0.50–2.44) | **1.60 (1.13–2.27)** | **1.58 (1.17–2.13)** | **1.75 (1.28–2.40)** | **1.64 (1.12–2.41)** |
| ≥40 | 1.07 (0.15–7.75) | **2.28 (1.11–4.67)** | **2.20 (1.52–3.18)** | **1.86 (1.35–2.58)** | **1.76 (1.24–2.51)** |
| **Macrosomia** |  |  |  |  |  |
| <35 | Reference | 1.03 (0.81–1.31) | **1.38 (1.05–1.82)** | 1.06 (0.55–2.04) | 0.89 (0.28–2.82) |
| 35–39 | 1.00 (0.56–1.80) | 1.19 (0.91–1.56) | **1.23 (1.01–1.54)** | **1.22 (1.00–1.55)** | 1.26 (0.94–1.69) |
| ≥40 | 1.15 (0.28–4.69) | 1.10 (0.55–2.20) | **1.39 (1.02–1.90)** | **1.35 (1.05–1.73)** | **1.36 (1.03–1.78)** |

^a^ Data are relative risk with 95% confidence intervals, estimated by using multiple imputation data. Adjusted for paternal factors including occupation, smoking and alcohol consumption, and maternal factors including delivery year, ethnicity, education, occupation, annual household income, gestational age at enrolment, pre-pregnancy BMI, smoking and alcohol consumption within 6 months prior to pregnancy, parity, and method of conception.

# Supplementary Table 6–8. Sensitivity analyses of associations of paternal age with perinatal outcomes among women with spontaneous conception

**Supplementary Table 6. Associations of paternal age with maternal outcomes among women with spontaneous conception** ^a^

|  | **Paternal age (years)** | | | | |
| --- | --- | --- | --- | --- | --- |
| **Maternal outcomes** | **<30** | **30–34** | **35–39** | **40–44** | **≥45** |
| **Gestational diabetes mellitus** |  |  |  |  |  |
| Unadjusted | Reference | 1.43 (1.20–1.71) | 1.75 (1.49–2.06) | 2.09 (1.77–2.47) | 2.04 (1.69–2.46) |
| Adjusted | Reference | **1.28 (1.07–1.54)** | **1.32 (1.09–1.60)** | **1.43 (1.17–1.75)** | **1.35 (1.08–1.69)** |
| **Hypertensive disorders of pregnancy** |  |  |  |  |  |
| Unadjusted | Reference | 1.61 (1.20–2.16) | 1.82 (1.38–2.38) | 2.08 (1.57–2.76) | 2.15 (1.57–2.94) |
| Adjusted | Reference | 1.13 (0.83–1.53) | 0.94 (0.68–1.30) | 0.97 (0.69–1.35) | 0.98 (0.68–1.41) |
| **Preeclampsia** |  |  |  |  |  |
| Unadjusted | Reference | 2.08 (1.29–3.33) | 2.20 (1.41–3.43) | 2.7 (1.71–4.26) | 2.87 (1.75–4.70) |
| Adjusted | Reference | 1.48 (0.91–2.41) | 1.20 (0.72–2.00) | 1.28 (0.75–2.17) | 1.35 (0.76–2.41) |
| **Placenta accreta** **spectrum disorder** |  |  |  |  |  |
| Unadjusted | Reference | 1.50 (1.00–2.26) | 2.79 (1.93–4.04) | 3.51 (2.41–5.11) | 3.19 (2.12–4.80) |
| Adjusted | Reference | 1.13 (0.74–1.73) | 1.17 (0.76–1.81) | 1.35 (0.87–2.12) | 1.34 (0.83–2.16) |
| **Placenta previa** |  |  |  |  |  |
| Unadjusted | Reference | 1.77 (1.22–2.57) | 2.52 (1.79–3.56) | 3.16 (2.23–4.48) | 2.65 (1.80–3.91) |
| Adjusted | Reference | 1.29 (0.88–1.89) | 1.27 (0.85–1.88) | 1.48 (0.98–2.22) | 1.26 (0.80–1.97) |
| **Cesarean delivery** |  |  |  |  |  |
| Unadjusted | Reference | 1.28 (1.14–1.43) | 1.69 (1.52–1.87) | 1.79 (1.61–2.00) | 1.86 (1.65–2.10) |
| Adjusted | Reference | **1.17 (1.04–1.31)** | **1.27 (1.13–1.44)** | **1.27 (1.11–1.45)** | **1.32 (1.14–1.53)** |
| **Postpartum hemorrhage** |  |  |  |  |  |
| Unadjusted | Reference | 1.86 (1.26–2.76) | 2.53 (1.76–3.65) | 3.11 (2.14–4.51) | 2.99 (1.99–4.49) |
| Adjusted | Reference | 1.28 (0.85–1.92) | 1.41 (0.93–2.13) | **1.59 (1.03–2.45)** | 1.56 (0.98–2.49) |
| **Adverse maternal outcome** |  |  |  |  |  |
| Unadjusted | Reference | 1.29 (1.17–1.42) | 1.55 (1.42–1.69) | 1.64 (1.49–1.80) | 1.68 (1.51–1.87) |
| Adjusted | Reference | **1.19 (1.07–1.31)** | **1.25 (1.12–1.39)** | **1.26 (1.13–1.41)** | **1.29 (1.14–1.47)** |

^a^ Data are relative risk with 95% confidence intervals. Adjusted for paternal factors including occupation, smoking and alcohol consumption, and maternal factors including delivery year, age, ethnicity, education, occupation, annual household income, gestational age at enrolment, pre-pregnancy BMI, smoking and alcohol consumption within 6 months prior to pregnancy, parity, and method of conception.

**Supplementary Table 7. Associations of paternal age with offspring outcomes among women with spontaneous conception** ^a^

|  | **Paternal age (years)** | |  |  |  |
| --- | --- | --- | --- | --- | --- |
| **Offspring outcomes** | **<30** | **30–34** | **35–39** | **40–44** | **≥45** |
| **Preterm birth** |  |  |  |  |  |
| Unadjusted | Reference | 1.53 (1.16–2.03) | 1.90 (1.47–2.46) | 2.31 (1.77–3.00) | 2.07 (1.53–2.78) |
| Adjusted | Reference | 1.29 (0.99–1.72) | 1.19 (0.88–1.62) | **1.33 (1.00–1.83)** | 1.31 (0.92–1.86) |
| **Large for gestational age** ^b^ |  |  |  |  |  |
| Unadjusted | Reference | 1.22 (0.99–1.52) | 1.49 (1.23–1.81) | 1.51 (1.23–1.85) | 1.53 (1.22–1.92) |
| Adjusted | Reference | 1.09 (0.87–1.37) | **1.29 (1.02–1.63)** | **1.31 (1.02–1.67)** | **1.38 (1.04–1.81)** |
| **Small for gestational age** ^b^ |  |  |  |  |  |
| Unadjusted | Reference | 0.93 (0.67–1.27) | 1.01 (0.76–1.34) | 1.00 (0.74–1.35) | 0.80 (0.55–1.17) |
| Adjusted | Reference | 0.98 (0.70–1.38) | 1.02 (0.71–1.45) | 1.01 (0.69–1.49) | 0.84 (0.53–1.33) |
| **Macrosomia** |  |  |  |  |  |
| Unadjusted | Reference | 1.25 (1.01–1.54) | 1.66 (1.37–2.00) | 1.71 (1.40–2.08) | 1.76 (1.41–2.21) |
| Adjusted | Reference | 1.12 (0.90–1.40) | **1.36 (1.08–1.71)** | **1.36 (1.06–1.73)** | **1.46 (1.11–1.91)** |
| **Congenital anomaly** |  |  |  |  |  |
| Unadjusted | Reference | 1.70 (1.00–2.90) | 2.19 (1.33–3.59) | 2.64 (1.59–4.39) | 2.79 (1.60–4.84) |
| Adjusted | Reference | 1.21 (0.69–2.12) | 1.08 (0.61–1.91) | 1.19 (0.66–2.16) | 1.16 (0.61–2.21) |
| **Adverse offspring outcome** |  |  |  |  |  |
| Unadjusted | Reference | 1.11 (0.99–1.24) | 1.09 (0.98–1.21) | 1.10 (0.99–1.23) | 1.16 (1.03–1.32) |
| Adjusted | Reference | 1.09 (0.93–1.28) | **1.20 (1.02–1.41)** | **1.22 (1.03–1.45)** | 1.19 (0.98–1.45) |

^a^ Data are relative risk with 95% confidence intervals. Adjusted for paternal factors including occupation, smoking and alcohol consumption, and maternal factors including delivery year, age, ethnicity, education, occupation, annual household income, gestational age at enrolment, pre-pregnancy BMI, smoking and alcohol consumption within 6 months prior to pregnancy, parity, and method of conception

^b^ Among a total of 16 114 singleton women analyzed in this study, there were 2416 women (15.0%) with missing information on birth weight, neonatal sex, or gestational age at delivery who were not included when performing the association analyses.

**Supplementary Table 8. Joint associations of paternal age and maternal age with the adverse perinatal outcome among women with spontaneous conception** ^a^

|  | **Paternal age (years)** | | | | |
| --- | --- | --- | --- | --- | --- |
| **Maternal age (years)** | **<30** | **30–34** | **35–39** | **40–44** | **≥45** |
| **Gestational diabetes mellitus** |  |  |  |  |  |
| <35 | Reference | **1.30 (1.06–1.60)** | **1.41 (1.10–1.82)** | **2.21 (1.39–3.51)** | 1.67 (0.68–4.09) |
| 35–39 | **1.67 (1.07–2.62)** | **1.91 (1.52–2.40)** | **1.91 (1.57–2.33)** | **2.10 (1.71–2.58)** | **1.90 (1.47–2.45)** |
| ≥40 | 2.48 (0.91–6.77) | **2.60 (1.49–4.54)** | **2.34 (1.79–3.08)** | **2.39 (1.92–2.97)** | **2.38 (1.88–3.01)** |
| **Cesarean delivery** |  |  |  |  |  |
| <35 | Reference | **1.20 (1.05–1.37)** | **1.45 (1.24–1.71)** | **1.52 (1.08–2.16)** | **1.78 (1.02–3.10)** |
| 35–39 | **1.35 (1.01–1.80)** | **1.39 (1.20–1.63)** | **1.48 (1.30–1.69)** | **1.48 (1.29–1.70)** | **1.50 (1.27–1.77)** |
| ≥40 | **2.67 (1.49–4.78)** | **1.62 (1.09–2.40)** | **1.59 (1.33–1.90)** | **1.65 (1.43–1.91)** | **1.76 (1.51–2.06)** |
| **Preterm birth** |  |  |  |  |  |
| <35 | Reference | 1.24 (0.90–1.71) | 1.17 (0.78–1.75) | 1.02 (0.37–2.83) | 1.76 (0.43–7.26) |
| 35–39 | 1.13 (0.51–2.49) | **1.69 (1.18–2.42)** | **1.54 (1.13–2.10)** | **1.86 (1.35–2.57)** | **1.70 (1.13–2.56)** |
| ≥40 | 1.30 (0.18–9.50) | **2.53 (1.18–5.39)** | **2.17 (1.47–3.21)** | **1.90 (1.35–2.66)** | **1.99 (1.38–2.88)** |
| **Macrosomia** |  |  |  |  |  |
| <35 | Reference | 1.07 (0.84–1.36) | **1.58 (1.20–2.09)** | 1.27 (0.66–2.44) | 1.28 (0.40–4.04) |
| 35–39 | 1.00 (0.55–1.84) | 1.23 (0.93–1.63) | **1.32 (1.05–1.66)** | **1.34 (1.04–1.71)** | **1.47 (1.08–1.99)** |
| ≥40 | 0.73 (0.10–5.29) | 1.01 (0.44–2.33) | **1.49 (1.08–2.06)** | **1.49 (1.15–1.94)** | **1.59 (1.2–2.12)** |

^a^ Data are relative risk with 95% confidence intervals. Adjusted for paternal factors including occupation, smoking and alcohol consumption, and maternal factors including delivery year, ethnicity, education, occupation, annual household income, gestational age at enrolment, pre-pregnancy BMI, smoking and alcohol consumption within 6 months prior to pregnancy, parity, and method of conception.

# Supplementary Table 9–11. Sensitivity analyses of associations of paternal age with perinatal outcomes using ordinal regression

**Supplementary Table 9. Associations of paternal age with maternal outcomes** ^a^

|  | **Paternal age (years)** | | | | |
| --- | --- | --- | --- | --- | --- |
| **Maternal outcomes** | **<30** | **30–34** | **35–39** | **40–44** | **≥45** |
| **Gestational diabetes mellitus** |  |  |  |  |  |
| Unadjusted | Reference | 1.61 (1.34–1.93) | 2.11 (1.78–2.49) | 2.65 (2.23–3.16) | 2.83 (2.33–3.43) |
| Adjusted | Reference | **1.37 (1.13–1.66)** | **1.44 (1.18–1.76)** | **1.58 (1.28–1.96)** | **1.56 (1.23–1.97)** |
| **Hypertensive disorders of pregnancy** |  |  |  |  |  |
| Unadjusted | Reference | 1.62 (1.22–2.15) | 2.01 (1.54–2.61) | 2.13 (1.63–2.80) | 2.42 (1.79–3.25) |
| Adjusted | Reference | 1.05 (0.77–1.42) | 0.91 (0.67–1.25) | 0.85 (0.61–1.18) | 0.91 (0.64–1.31) |
| **Preeclampsia** |  |  |  |  |  |
| Unadjusted | Reference | 1.99 (1.27–3.10) | 2.39 (1.58–3.64) | 2.70 (1.76–4.14) | 2.78 (1.75–4.42) |
| Adjusted | Reference | 1.33 (0.83–2.12) | 1.20 (0.74–1.94) | 1.18 (0.72–1.96) | 1.18 (0.69–2.03) |
| **Placenta accreta** **spectrum disorder** |  |  |  |  |  |
| Unadjusted | Reference | 1.60 (1.09–2.35) | 3.10 (2.18–4.40) | 3.77 (2.64–5.39) | 3.70 (2.52–5.42) |
| Adjusted | Reference | 1.10 (0.73–1.65) | 1.14 (0.75–1.73) | 1.28 (0.84–1.97) | 1.28 (0.81–2.02) |
| **Placenta previa** |  |  |  |  |  |
| Unadjusted | Reference | 1.87 (1.30–2.68) | 2.80 (2.00–3.90) | 3.36 (2.39–4.72) | 3.14 (2.17–4.54) |
| Adjusted | Reference | 1.31 (0.90–1.90) | 1.30 (0.89–1.91) | 1.46 (0.98–2.17) | 1.35 (0.88–2.07) |
| **Cesarean delivery** |  |  |  |  |  |
| Unadjusted | Reference | 1.54 (1.34–1.78) | 2.85 (2.50–3.24) | 3.40 (2.96–3.90) | 4.16 (3.52–4.90) |
| Adjusted | Reference | **1.28 (1.10–1.49)** | **1.53 (1.30–1.80)** | **1.53 (1.28–1.82)** | **1.79 (1.46–2.19)** |
| **Postpartum hemorrhage** |  |  |  |  |  |
| Unadjusted | Reference | 2.02 (1.42–2.87) | 2.55 (1.83–3.55) | 3.06 (2.18–4.28) | 2.79 (1.93–4.03) |
| Adjusted | Reference | 1.28 (0.88–1.87) | 1.40 (0.95–2.06) | **1.56 (1.04–2.33)** | 1.39 (0.90–2.16) |
| **Adverse maternal outcome** |  |  |  |  |  |
| Unadjusted | Reference | 1.80 (1.57–2.07) | 3.44 (3.03–3.91) | 4.51 (3.91–5.21) | 5.29 (4.41–6.36) |
| Adjusted | Reference | **1.39 (1.20–1.62)** | **1.67 (1.42–1.97)** | **1.79 (1.49–2.16)** | **1.97 (1.57–2.46)** |

^a^ Data are relative risk with 95% confidence intervals, estimated by using ordinal regression. Adjusted for paternal factors including occupation, smoking and alcohol consumption, and maternal factors including delivery year, age, ethnicity, education, occupation, annual household income, gestational age at enrolment, pre-pregnancy BMI, smoking and alcohol consumption within 6 months prior to pregnancy, parity, and method of conception.

**Supplementary Table 10. Associations of paternal age with offspring outcomes** ^a^

|  | **Paternal age (years)** | |  |  |  |
| --- | --- | --- | --- | --- | --- |
| **Offspring outcomes** | **<30** | **30–34** | **35–39** | **40–44** | **≥45** |
| **Preterm birth** |  |  |  |  |  |
| Unadjusted | Reference | 1.63 (1.24–2.15) | 2.13 (1.65–2.76) | 2.49 (1.91–3.23) | 2.13 (1.59–2.86) |
| Adjusted | Reference | **1.33 (1.00–1.78)** | 1.29 (0.95–1.74) | **1.39 (1.01–1.90)** | 1.29 (0.91–1.83) |
| **Large for gestational age** ^b^ |  |  |  |  |  |
| Unadjusted | Reference | 1.26 (1.01–1.57) | 1.60 (1.31–1.95) | 1.62 (1.31–1.99) | 1.56 (1.23–1.98) |
| Adjusted | Reference | 1.08 (0.85–1.36) | 1.27 (0.99–1.62) | 1.26 (0.97–1.63) | 1.25 (0.94–1.67) |
| **Small for gestational age** ^b^ |  |  |  |  |  |
| Unadjusted | Reference | 0.95 (0.70–1.29) | 1.02 (0.77–1.34) | 0.91 (0.68–1.23) | 0.83 (0.59–1.18) |
| Adjusted | Reference | 1.01 (0.72–1.41) | 1.10 (0.77–1.56) | 0.98 (0.67–1.44) | 0.94 (0.61–1.45) |
| **Macrosomia** |  |  |  |  |  |
| Unadjusted | Reference | 1.30 (1.05–1.61) | 1.81 (1.49–2.20) | 1.86 (1.52–2.28) | 1.84 (1.47–2.32) |
| Adjusted | Reference | 1.13 (0.90–1.41) | **1.34 (1.06–1.69)** | **1.31 (1.02–1.67)** | **1.33 (1.01–1.75)** |
| **Congenital anomaly** |  |  |  |  |  |
| Unadjusted | Reference | 1.77 (1.06–2.98) | 2.36 (1.46–3.82) | 2.72 (1.66–4.46) | 3.03 (1.79–5.14) |
| Adjusted | Reference | 1.17 (0.68–2.03) | 1.04 (0.60–1.82) | 1.12 (0.63–2.00) | 1.11 (0.60–2.06) |
| **Adverse offspring outcome** |  |  |  |  |  |
| Unadjusted | Reference | 1.30 (1.11–1.54) | 1.83 (1.57–2.12) | 1.90 (1.63–2.22) | 1.83 (1.54–2.19) |
| Adjusted | Reference | 1.11 (0.94–1.32) | **1.26 (1.05–1.51)** | **1.23 (1.01–1.49)** | 1.20 (0.97–1.49) |

^a^ Data are relative risk with 95% confidence intervals, estimated by using ordinal regression. Adjusted for paternal factors including occupation, smoking and alcohol consumption, and maternal factors including delivery year, age, ethnicity, education, occupation, annual household income, gestational age at enrolment, pre-pregnancy BMI, smoking and alcohol consumption within 6 months prior to pregnancy, parity, and method of conception.

^b^ Among a total of 16 114 singleton women analyzed in this study, there were 2416 women (15.0%) with missing information on birth weight, neonatal sex, or gestational age at delivery who were not included when performing the association analyses.

**Supplementary Table 11. Joint associations of paternal age and maternal age with the adverse perinatal outcome** ^a^

|  | **Paternal age (years)** | | | | |
| --- | --- | --- | --- | --- | --- |
| **Maternal age (years)** | **<30** | **30–34** | **35–39** | **40–44** | **≥45** |
| **G****estational diabetes mellitus** |  |  |  |  |  |
| <35 | Reference | **1.39 (1.12–1.73)** | **1.58 (1.22–2.06)** | **2.82 (1.66–4.79)** | 2.13 (0.91–4.99) |
| 35–39 | **1.86 (1.12–3.08)** | **2.27 (1.78–2.90)** | **2.34 (1.90–2.88)** | **2.64 (2.12–3.29)** | **2.35 (1.80–3.06)** |
| ≥40 | **4.06 (1.32–12.5)** | **4.23 (2.35–7.59)** | **3.17 (2.36–4.26)** | **3.19 (2.52–4.02)** | **3.52 (2.74–4.51)** |
| **Cesarean delivery** |  |  |  |  |  |
| <35 | Reference | **1.31 (1.11–1.55)** | **1.81 (1.48–2.22)** | **2.04 (1.25–3.32)** | 2.02 (0.93–4.38) |
| 35–39 | **1.53 (1.00–2.35)** | **1.70 (1.39–2.08)** | **1.94 (1.64–2.28)** | **1.95 (1.63–2.32)** | **2.07 (1.65–2.60)** |
| ≥40 | **24.63 (3.16–192.29)** | **2.43 (1.30–4.56)** | **2.53 (1.90–3.36)** | **3.00 (2.45–3.68)** | **3.97 (3.14–5.00)** |
| **Preterm birth** |  |  |  |  |  |
| <35 | Reference | 1.29 (0.94–1.78) | 1.25 (0.85–1.84) | 0.86 (0.30–2.46) | 2.56 (0.85–7.70) |
| 35–39 | 1.04 (0.46–2.38) | **1.69 (1.18–2.41)** | **1.62 (1.19–2.20)** | **1.86 (1.35–2.57)** | **1.68 (1.13–2.49)** |
| ≥40 | 1.03 (0.13–8.30) | **2.53 (1.18–5.43)** | **2.36 (1.59–3.51)** | **1.99 (1.42–2.79)** | **1.89 (1.31–2.73)** |
| **Macrosomia** |  |  |  |  |  |
| <35 | Reference | 1.07 (0.84–1.38) | **1.62 (1.22–2.16)** | 1.13 (0.56–2.28) | 0.83 (0.24–2.82) |
| 35–39 | 1.04 (0.55–1.94) | 1.31 (0.99–1.75) | **1.38 (1.09–1.76)** | **1.38 (1.07–1.78)** | **1.44 (1.05–1.96)** |
| ≥40 | 1.27 (0.27–5.88) | 1.11 (0.52–2.37) | **1.64 (1.17–2.29)** | **1.57 (1.20–2.05)** | **1.60 (1.19–2.14)** |

^a^ Data are relative risk with 95% confidence intervals, estimated by using ordinal regression. Adjusted for paternal factors including occupation, smoking and alcohol consumption, and maternal factors including delivery year, ethnicity, education, occupation, annual household income, gestational age at enrolment, pre-pregnancy BMI, smoking and alcohol consumption within 6 months prior to pregnancy, parity, and method of conception.
